# Haemodynamic monitoring and management during non-cardiac surgery: a survey among German anaesthesiologists

**DOI:** 10.1007/s10877-025-01284-0

**Published:** 2025-03-22

**Authors:** Benjamin Vojnar, Patrick Achenbach, Moritz Flick, Daniel Reuter, Michael Sander, Bernd Saugel, Ann-Kristin Schubert, Christine Gaik

**Affiliations:** 1 Department of Anesthesiology and Intensive Care Medicine, University Hospital Giessen and Marburg, Campus Marburg and Philipps-University of Marburg, Marburg, Germany; 2 Department of Anesthesiology, Intensive Care and Pain Medicine, Klinikum Dortmund, Dortmund, Germany; 3 Department of Anesthesiology, Center of Anesthesiology and Intensive Care Medicine, University Medical Center Hamburg-Eppendorf, Hamburg, Germany; 4 Department of Anesthesia and Intensive Care, University Hospital Rostock, Rostock, Germany; 5 Department of Anaesthesiology, Intensive Care Medicine and Pain Therapy, Justus Liebig University Giessen University Hospital Giessen, UKGM, Giessen, Germany

**Keywords:** Advanced haemodynamic monitoring, Hypotension, Perioperative medicine, Blood pressure measurement

## Abstract

In 2023, the first German guideline on intraoperative haemodynamic monitoring and management for adults having non-cardiac surgery was published. The aim of this survey was to identify how anaesthetists in Germany managed intraoperative haemodynamics and blood pressure before its publication. In September to October 2023, members of the German Society of Anaesthesiology and Intensive Care Medicine (DGAI) were invited via email to participate in this anonymous online survey. Thirty-one questions covered demographics, clinical experience, approaches to perioperative blood pressure measurement and common thresholds, as well as the use of advanced haemodynamic monitoring and its potential therapeutic implications. 1,079 fully completed questionnaires were included in the analysis. When intermittent oscillometry was used to measure blood pressure, a 3-minute interval was usually applied during induction of anaesthesia (42%; 451/1,079). For invasive blood pressure monitoring, more than half (53%; 574/1,079) inserted an arterial line after induction of anaesthesia. Nearly all (94%; 1,012/1,079) focused on the mean arterial pressure for blood pressure monitoring, with a large majority (77%; 779/1012) considering values below 60–65 mmHg to be critically low. Intraoperative hypotension was managed based on an internal protocol by only 21% (223/1,079). Regarding advanced haemodynamic monitoring, 43% (459/1,079) frequently used pulse contour analysis, while 67% (721/1,079) reported that monitors with finger-cuff technology were not available in their department. 47% (504/1,079) cited a lack of experience as one of the main reasons for the infrequent use of cardiac output monitoring. This survey among DGAI members provides important insights into current practices of haemodynamic monitoring and management prior to the publication of the recent German guideline on ‘Intraoperative haemodynamic monitoring and management of adults having non-cardiac surgery’.

**Supplementary Information:**

The online version contains supplementary material available at 10.1007/s10877-025-01284-0.

## Introduction

Basic haemodynamic monitoring is a key component of perioperative care and ensures patient safety. Advanced haemodynamic monitoring including measuring cardiac output (CO)– the primary haemodynamic determinant of oxygen delivery– may provide critical insights into the causes of haemodynamic instability and guide therapy with fluids, vasopressors, and inotropes.

Over the past decades, many minimally invasive and non-invasive methods for advanced haemodynamic monitoring have been developed [1–3]. Consequently, highly invasive methods, such as pulmonary artery catheterisation or transpulmonary thermodilution, are nowadays rarely used in non-cardiac surgery patients. However, it largely remains unclear how intraoperative haemodynamics are monitored and managed. In 2023, the first German guideline for intraoperative haemodynamic monitoring and management of adults having non-cardiac surgery was published [4]. This survey aimed to explore how anaesthesiologists in Germany performed intraoperative haemodynamic monitoring and management prior to guideline publication.

## Methods

We conducted an anonymous web-based survey among members of the German Society of Anaesthesiology and Intensive Care Medicine (DGAI). The survey was created using an online platform (SurveyMonkey, San Mateo, California, USA). An invitation to participate in the survey was sent to 23,229 DGAI members by email on September 7, 2023, followed by a reminder email on September 25, 2023.

The survey consisted of 31 questions and a brief introduction outlining the rationale and aim of the survey. Participation in the survey was voluntary, and no financial or other incentives were offered. Web cookies were used to limit responses to one per participant. The order of the questions was the same for all participants.

The 31 questions included 29 multiple-choice questions (23 with single-answer option and six with multiple-answer option) and two matrix questions with answers based on a frequency rating scale. Fourteen questions allowed the answer ‘other’, two questions were skip logic questions, and one question allowed free-text responses.

The first three questions asked about the respondents’ demographics, clinical experience, position, and current hospital. Seven questions focused on perioperative blood pressure measurement and six questions on the use of advanced haemodynamic monitoring and its potential therapeutic consequences. In another six questions, respondents were asked about hypotension during induction of anaesthesia and during surgery. Four questions focused on blood pressure intervention thresholds. Finally, five questions were asked about fluid therapy.

We only analyzed fully completed surveys. Although respondents were required to answer each question before proceeding, it was possible to skip individual questions. We therefore report the number of respondents for each question. We provide the absolute and relative number of responses. All responses received were used as a random sample, and no sample size calculation was performed.

Data were managed and analyzed using Excel (Microsoft; Redmond, Washington, USA).

## Results

Out of the 23,229 DGAI members who received the invitation email, 5,386 opened it, and 1,237 participated in the survey (5% of all DGAI members and 23% of those who opened the email). A total of 158 surveys were not completely answered and excluded from the analysis. Therefore, we analyzed 1,079 fully completed surveys. Data on the respondents’ demographics, clinical experience, positions, and hospital settings are shown in Table 1.

**Table 1:** Data on the respondents’ demographics, clinical experience, positions, and current hospital

	*N*	
Professional experience		
< 5 years	116	11%
5–10 years	221	21%
11–20 years	302	28%
> 20 years	440	41%
Job position		
Resident	173	16%
Consultant	227	21%
Specialised Consultant	46	4%
Senior consultant	473	44%
Chief physician	141	13%
Other position	19	2%
Workplace / Hospital of the respondents		
Primary care hospitals (≤ 299 beds)	180	17%
Standard care hospitals (300–499 beds)	213	20%
Specialized care hospitals (500–799 beds)	218	20%
Maximum / University care hospitals (≥ 800 beds)	416	39%
Outpatient anaesthesia / Ambulatory surgery center	10	1%
Other workplace	42	4%

### Perioperative blood pressure measurement

**Fig. 1:**
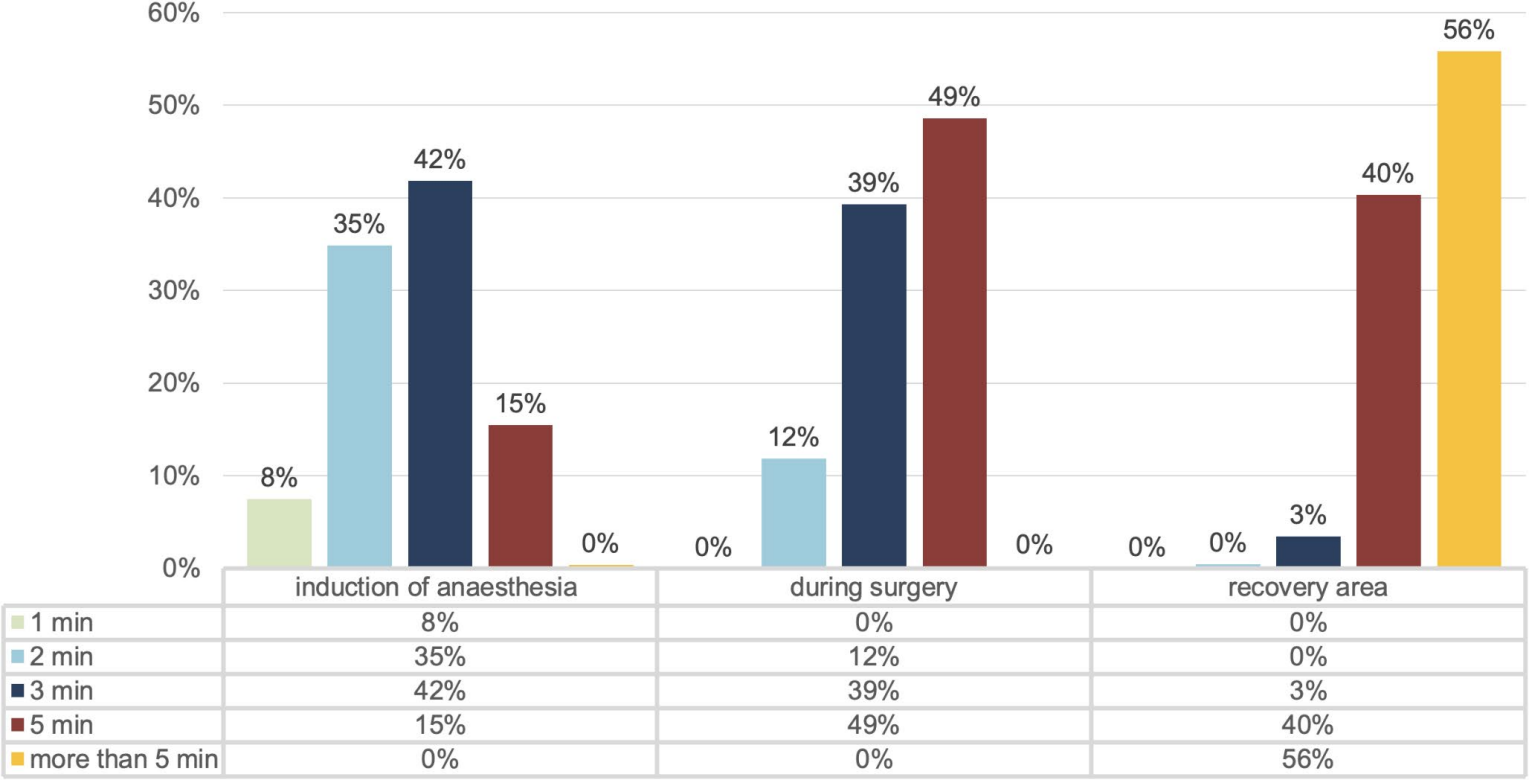
Measurement intervals for intermittent oscillometric blood pressure monitoring during induction of anaesthesia, during surgery, and in the recovery area (1,079 responses each)

When intermittent oscillometry is used to measure blood pressure, most respondents use a measurement interval of three minutes during induction of anaesthesia (42%; 451/1,079), of five minutes during surgery (49%; 524/1,079), and of more than five minutes (56%; 602/1,079) in the post-anaesthesia care unit (see Fig. 1).

**Fig. 2:**
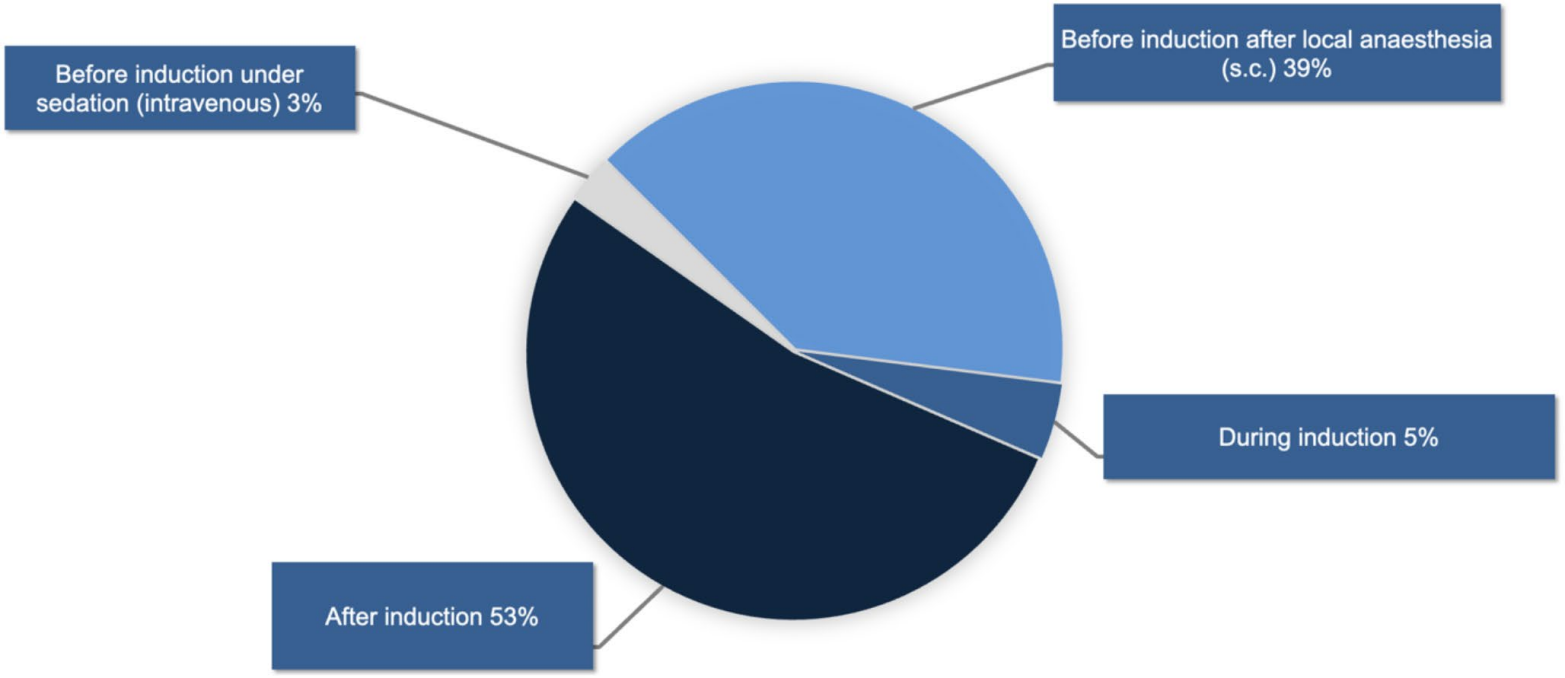
Timepoint of arterial catheter insertion (1,079 responses)

When an arterial catheter is used to measure blood pressure, 42% of the respondents (456/1,079) routinely insert the arterial catheter before induction of anaesthesia, and 53% of the respondents (574/1,079) routinely insert it after induction of anaesthesia, (see Fig. 2).

Nearly all respondents (99%, 1,073/1,079) insert arterial catheters in the radial artery. 14% of the respondents (153/1,079) “always” use ultrasound for arterial catheter insertion, while 24% (257/1,079) use it only after the “first failed attempt”, 28% (303/1,079) after “multiple failed attempts”, 32% (344/1,079) use ultrasound “sometimes”, and 2% (22/1,079) “never” use ultrasound for arterial catheter insertion.

To routinely check the quality of the arterial blood pressure waveform (e.g., for over- and underdamping), 74% of the respondents (795/1,079) visually inspect the waveform, and 20% (214/1,079) perform a square wave test (fast-flush test). 5% of the respondents (59/1,079) do not routinely check the quality of the blood pressure waveform.

Among patients undergoing surgery in sitting positions, 43% of the respondents (466/1,079) position the pressure transducer at the level of the ear canal, while 54% (574/1,079) position it at the level of the heart.

**Fig. 3:**
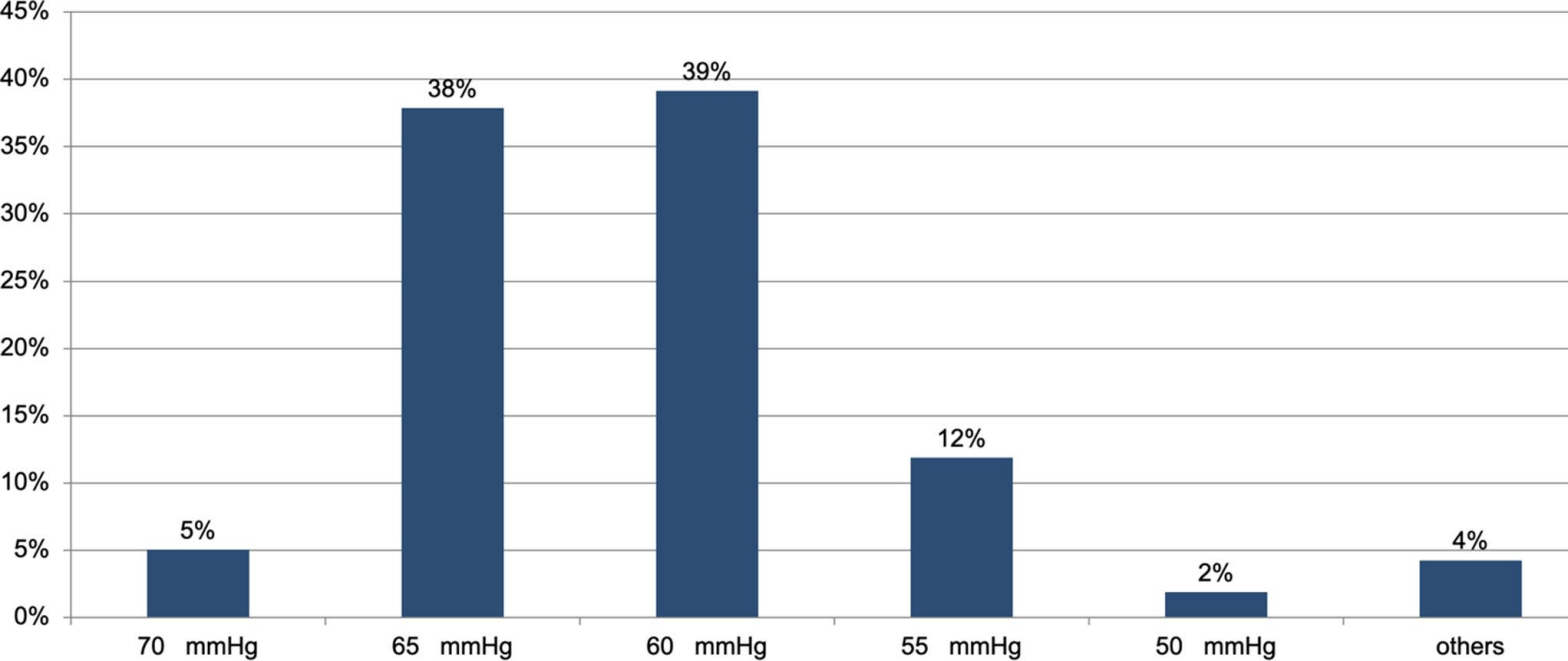
The figure shows which mean arterial pressure values the participants considered to be critically low (1,079 responses)

Nearly all respondents (94%; 1,012/1,079) use mean arterial pressure to guide blood pressure management– only 6% (66/1,079) use systolic blood pressure. Diastolic blood pressure is almost never used (0.1%; 1/1,079). Of the 1012 respondents who use mean arterial pressure to guide blood pressure management, 39% (396/1012) consider a mean arterial pressure of 60 mmHg as “critically low”, while 38% (383/1012) define a mean arterial pressure of 65 mmHg as “critically low” (see Fig. 3). When asked how intraoperative hypotension is treated in their institution, more than half (56%, 607/1,079) of the respondents stated that they treat it at their own discretion. 23% (249/1,079) follow the instructions of a consultant anaesthesiologist, and 21% (223/1,079) adhere to an institutional protocol.

### Advanced haemodynamic monitoring

When asked about factors that influence their decision to use advanced haemodynamic monitoring, respondents ‘always/frequently’ consider the patients’ comorbidities (92%, 993/1,079), the surgical risk (88%, 951/1,079), the expected blood loss (85%, 917/1,079), or the American Society of Anesthesiologists physical status class system (70%, 758/1,079). The duration of surgery is considered as a criterium “occasionally” by 52% (563/1,079) of respondents and “rarely/never” by 16% (174/1,079) of respondents. The most commonly used methods for advanced haemodynamic monitoring in the respondents’ hospitals are shown in Table 2.

**Table 2:** Methods used for advanced haemodynamic monitoring in their hospitals and how often they use it. Data are shown as percentage with absolute numbers

	Frequently		Rarely		Never		Technology	
							Not available	
Pulse contour analysis (*n* = 1,079)	43%	(459)	39%	(422)	7%	(72)	12%	(126)
TTE (*n* = 1,079)	35%	(377)	44%	(480)	15%	(158)	6%	(64)
CVP monitoring (*n* = 1,079)	32%	(347)	43%	(466)	22%	(242)	2%	(24)
TOE (*n* = 1,079)	25%	(275)	48%	(515)	17%	(179)	10%	(110)
Transpulmonary thermodilution (*n* = 1,079)	25%	(273)	49%	(532)	12%	(129)	13%	(145)
Pulmonary artery catheter (*n* = 1,079)	5%	(52)	37%	(400)	29%	(310)	29%	(317)
Finger cuff method (*n* = 1,079)	2%	(21)	11%	(120)	20%	(217)	67%	(721)
Bioreactance / bioimpedance technology (*n* = 1,079)	1%	(15)	7%	(77)	16%	(173)	75%	(814)
Esophageal Doppler (*n* = 1,079)	1%	(11)	13%	(136)	27%	(294)	59%	(638)

CVP = Central venous pressure; TOE = Transesophageal echocardiography; TTE = Transthoracic echocardiography

### Perspectives of haemodynamic monitoring

**Fig. 4:**
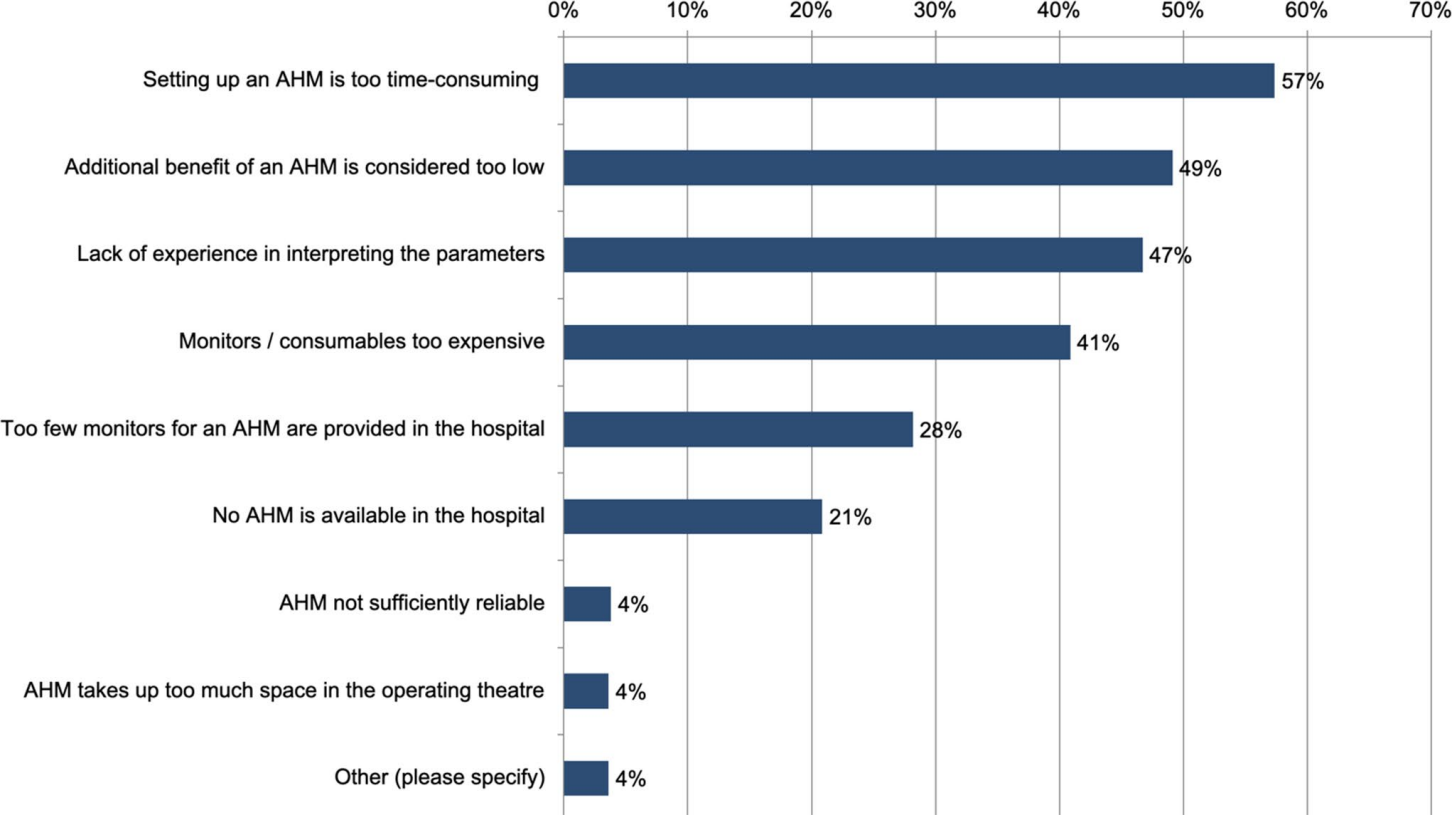
Reasons why haemodynamic monitoring is not used during high-risk non-cardiac surgery (1,079 responses). AHM = Advanced haemodynamic monitoring

When asked about their opinion, why institutions do not use advanced haemodynamic monitoring in high-risk non-cardiac surgery patients, 57% of the respondents (619/1,079) cited the time-consuming setup, 49% (530/1,079) felt the added value is too low, and 47% (504/1,079) reported a lack of experience in interpreting the parameters. 41% (441/1,079) pointed the high cost of monitors and consumables, while 28% (304/1,079) noted insufficient availabilityof monitors in their hospital (see Fig. 4).

62% (660/1,079) and 34% (370/1,079) of the respondents think that advanced haemodynamic monitoring ‘sometimes’ or ‘(almost) always’ improves patient care in the operating room.

### Fluid responsiveness

**Fig. 5:**
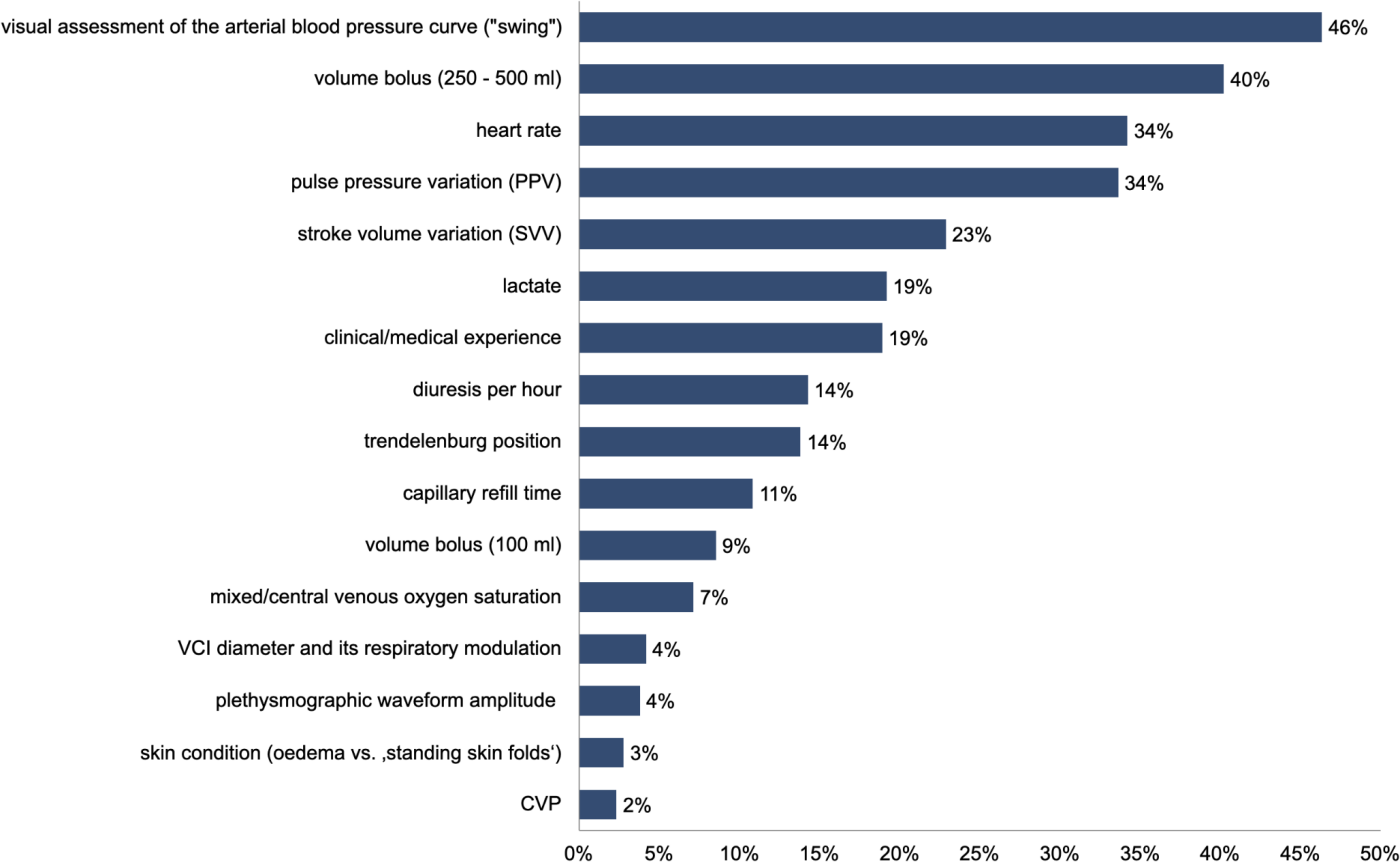
Methods used to assess fluid responsiveness. AHM = Advanced haemodynamic monitoring; CVP = Central venous pressure; VCI = Inferior vena cava

When asked how they preferably assess fluid responsiveness in patients with sinus rhythm and controlled mechanical ventilation, 46% of the respondents (500/1,079) use visual interpretation of the arterial blood pressure waveform (‘swing’), 40% (434/1,079) use a ‘fluid challenge’ (250–500 ml), and 34% (369/1,079) consider heart rate (see Fig. 5).

When performing fluid challenges, 97% of the respondents (1,051/1,079) use balanced crystalloid fluids.

## Discussion

This web-based survey among DGAI members provides insights into current practices of haemodynamic monitoring and management prior to the publication of the recent German guideline on ‘Intraoperative haemodynamic monitoring and management of adults having non-cardiac surgery’ [4]. The findings indicate that DGAI members surveyed largely follow international recommendations, such as general blood pressure thresholds, measurement intervals and indications for advanced haemodynamic monitoring. Protocols for haemodynamic management and blood pressure therapy appear to be standardised to a limited degree.

An association between perioperative hypotension and postoperative organ injury has been shown in large database studies [5–8]. Although there is no standardised definition of hypotension [9, 10], it is often described as a mean arterial pressure below 60–65 mmHg– a threshold associated with an increased risk of acute kidney and myocardial injury [10, 11]. In line with this, a large proportion of respondents manage blood pressure according to mean arterial pressure, considering values of 60–65 mmHg to be critically low.

In most patients, blood pressure is measured perioperatively by intermittent oscillometric monitoring. For induction of anaesthesia, many respondents measure blood pressure every two or three minutes [7, 12, 13]. During surgery, the most commonly reported measurement interval is longer– usually three or five minutes– presumably because anaesthesiologists acknowledge that the induction of anaesthesia is often associated with hypotension [13]. In the recovery area, when patients are awake after surgery, the measurement interval is often extended beyond five minutes– presumably because patients are more haemodynamically stable [14]. When intra-arterial blood pressure monitoring is indicated, more than half of the respondents insert arterial catheters only after induction of anaesthesia– probably assuming that this increases patient comfort. However, inserting the arterial catheter before induction of anaesthesia reduces hypotension [15] and similar procedures such as arterial puncture during cardiac catheterisation are routinely performed in awake patients. Survey respondents prefer the radial artery for arterial catheter insertion due to its safety and lower complications rates compared other arterial sites [16].

Almost all respondents indicated that ultrasound devices are available for haemodynamic monitoring. Despite this high availability, arterial catheterisation is rarely performed with ultrasound guidance, even though the benefits of this approach are well-documented [17–23].

Checking the quality of the arterial waveform is important, especially when using pulse wave analysis [24–27]. While about 75% of respondents visually assess the blood pressure curve to identify damping phenomena, only 20% use standardised tests [26, 28]. Notably, a recent survey of members of the European Society of Anaesthesiology and Intensive Care (ESAIC) reported that 50% of respondents employ a standardised fast-flush test [29]. There appear to be differences between the two professional groups.

In addition to continuous invasive blood pressure measurement, continuous non-invasive monitoring using a finger cuff can also help to reduce intraoperative hypotension [29, 30]. However, two-thirds of the respondents indicated that they do not have access to such monitoring systems. Only 2% reported using this technology frequently. At present, finger cuff technologies are not routinely used by the respondents.

In 2011 and 2021, 30% and 23% of members of the ESAIC stated that they have institutional haemodynamic treatment protocols [31, 32]. In this survey, participants were asked about the use of standardised protocols for the treatment of intraoperative hypotension. Only one in five respondents uses a standardised protocol to treat intraoperative hypotension, aligning with the 23% reported in a survey among ESAIC members. Although the ESAIC survey asked for a standardised treatment protocol for haemodynamic management, the therapy of blood pressure and flow among respondents appears to be less standardised. This could be due to the lack of evidence that a standardised treatment protocol reduces postoperative complications [33–35].

Without the use of protocols, the respondents in our survey usually base their treatment decisions on their personal expertise or on instructions from superiors. The analysis by level of professional experience shows that with increasing experience, a greater proportion of respondents rely on their personal clinical judgement to guide the treatment of hypotension, rather than adhering to protocols. These findings suggest that clinical experience plays a crucial role in treatment decisions, which, however, may lead to a considerable variability and low reproducibility in clinical practice [36].

The respondents use a variety of techniques and parameters to predict fluid responsiveness, which is consistent with the results of the survey of ESAIC members [37]. To estimate intraoperative volume responsiveness, most respondents primarily assess the ‘swing’ in the blood pressure waveform, which is a highly inaccurate method [38]. In addition, the performance of a volume challenge with 250–500 ml of crystalloid fluid is commonly used, and heart rate or pulse pressure variation is assessed.

According to current recommendations [39], respondents opt for advanced haemodynamic monitoring based on patient comorbidities, surgical risk, and expected blood loss. Although these considerations may seem justified, studies have consistently shown that advanced haemodynamic monitoring is still rarely used in surgical patients, even those at high risk [40, 41]. Respondents were asked to identify possible reasons.

A lack of experience in interpreting haemodynamic monitoring parameters was identified as a relevant barrier across all levels of professional experience. Clinicians with fewer years of experience appeared to be more affected, while those with greater experience reported this limitation less frequently. Overall, approximately half of the respondents cited insufficient experience in interpreting haemodynamic parameters as the primary reason for not using advanced haemodynamic monitoring in clinical practice. About one-fith of the respondents indicated that haemodynamic monitoring is not performed because it is not available. Notably, around 60% of respondents work in major or maximum care hospitals (including university hospitals). Consistent with this, 88% of respondents reported having pulse wave analysis monitors available in their hospital, but only 43% use them frequently. These findings highlight the need for training and education in perioperative haemodynamic monitoring.

Our study provides a comprehensive dataset based on 1,079 fully completed surveys.

But a potential limitation of this study is the response rate, with 5% of invited DGAI members participating. Not all DGAI members may have felt addressed, particularly those working exclusively in intensive care units (ICUs) or other non-anaesthetic settings. This may have affected both the overall response rate and introduced selection bias, as respondents could differ from non-respondents in terms of their clinical practice and attitudes towards haemodynamic monitoring. This survey was conducted among members of the DGAI, indicating that the results mainly reflect their approach to haemodynamic monitoring, and most of them were experienced physicians. Our results may not reflect the experiences of all anaesthetists, as the respondents are likely more engaged in haemodynamic monitoring and management. Since anaesthesiologists are primarily responsible for providing and monitoring haemodynamic management in Germany, the study focused on surveying this professional group. Although the role of nursing staff in haemodynamic management is undoubtedly important, our survey did not include questions about their influence, which is a slight limitation. Another potential limitation is the low proportion of respondents working in outpatient anaesthesia / ambulatory surgery center, accounting for only 1% of the survey population. This suggests that anaesthetists in this setting may be underrepresented. As with all medical surveys, discrepancies may exist between the self-reported data and actual clinical practice, which should be considered when interpreting the results. Finally, we would like to point out that, despite the use of cookies, we cannot rule out the possibility that some participants may have completed the questionnaire more than once.

We plan to repeat the survey several years after the publication of the German guideline on ‘Intraoperative haemodynamic monitoring and management of adults having non-cardiac surgery’ [4] to evaluate the impact of the guideline on clinical practice.

## Conclusion

In summary, our survey among DGAI members provides important insights into intraoperative haemodynamic monitoring and management prior to publication of the first German guideline on ‘Intraoperative haemodynamic monitoring and management of adults having non-cardiac surgery’ [4]. The survey indicates that the treatment of intraoperative hypotension rarely follows standardised protocols and is often still guided by personal clinical experience. Approximately 60% of the respondents work in maximum care hospitals, including university hospitals. While many have access to cardiac output monitors, only 43% use them regularly. A major hurdle appears to be the challenge of effectively integrating displayed haemodynamic parameters into clinical practice. In this context, the new guideline could provide valuable support among German anaesthesiologists.

## Electronic supplementary material

Below is the link to the electronic supplementary material.

Supplementary Material 1

## Data Availability

No datasets were generated or analysed during the current study.
