# Supplementary material for: Haemodynamic monitoring and management during non-cardiac surgery: a survey among German anaesthesiologists
Source: J Clin Monit Comput. 2025 Mar 22;39(5):853–61. doi: 10.1007/s10877-025-01284-0 (PMC12474607; doi:10.1007/s10877-025-01284-0)
Supplement: Supplementary file 1 — Supplementary Material 1 [file 10877_2025_1284_MOESM1_ESM.pdf]

Haemodynamic monitoring and management during non-cardiac surgery: a survey among German anaesthesiologists

The original questionnaire was created in German and now translated into English by us for publication. Some questions contained conditional logic: In certain cases, subsequent questions were only displayed if the previous answer met predefined criteria.

Q1. How many years have you been working in the field of anaesthesia?

Answer choices

a) Less than 5 years
b) 5 - 10 years
c) 11 - 20 years
d) More than 20 years

Q2. Please indicate your current position:

Answer choices

a) Resident
b) Consultant
c) Specialised Consultant
d) Senior consultant
e) Chief physician
f) Other position

Q3. How many beds does the hospital you work at have?

Answer choices

a) Primary care hospital (up to 299 beds)
b) Standard care hospital (300 to 499 beds)
c) Specialized care hospital (500 to 799 beds)
d) Maximum care hospital/university hospital (more than 800 beds)
e) Day surgery unit
f) Other (please specify)

Q4. What measurement interval do you use for oscillometric blood pressure monitoring (upper arm cuff) during the respective perioperative phases?

Answer choices	1 min	2 min	3 min	5 min	More than 5 min
Induction of anaesthesia					
Intraoperative					
In the recovery room					

Q5. At what point do you routinely establish invasive blood pressure monitoring?

Answer choices

a) Before induction of anaesthesia after local anaesthesia (s.c.)
b) Before induction of anaesthesia under sedation (i.v.)
c) During induction of anaesthesia
d) After induction of anaesthesia

Q6. Which puncture technique do you primarily use in the operating theatre for establishing invasive blood pressure monitoring ?
Answer choices

a) Seldinger technique (catheter over guidewire)
b) Direct puncture with an arterial cannula (similar to an intravenous cannula)

Q7. Which puncture site do you primarily choose in the operating theatre for establishing invasive blood pressure monitoring ?
Answer choices

a) Radial artery
b) Brachial artery
c) Femoral artery
d) Other (please specify)

Q8. Do you use ultrasound for establishing invasive blood pressure monitoring ?
Answer choices

a) Yes, always
b) Yes, after a single unsuccessful puncture
c) Yes, but only after multiple unsuccessful punctures
d) No, never
e) Occasionally (e.g., depending on puncture site, oedema, etc.)

Q9. How do you routinely assess the quality of the blood pressure waveform when using invasive blood pressure monitoring (e.g., for over- or underdamping)?
Answer choices

a) Visual inspection of the arterial blood pressure waveform
b) Square Wave Test/Fast Flush Test (eyeballing)
c) Square Wave Test/Fast Flush Test (calculated)
d) No routine quality assessment of the arterial blood pressure waveform
e) Other (please specify)

Q10. You are managing a patient in the sitting position (“beach-chair position”). Which position of the pressure transducer do you prefer in this situation?
Answer choices

a) At the level of the ear canal, fixed to the operating table
b) At the level of the heart, fixed to the operating table
c) At the level of the ear canal, fixed to an IV pole
d) At the level of the heart, fixed to an IV pole
e) Other (please specify)

Q11. Which of the following techniques do you currently use for advanced haemodynamic monitoring in your hospital?

Answer choices	Frequently	Rarely	Never	Technique not available
a) Bioreactance/Bioimpedance technology				
b) Fingercuff method				
c) Oesophageal Doppler				
d) Pulmonary artery catheter				
e) Pulse contour analysis				
f) Transpulmonary thermodilution				
g) Transoesophageal echocardiography				
h) Transthoracic echocardiography				
i) Central venous pressure monitoring				

Q12. According to which guidelines do you manage intraoperative hypotension in your hospital?

Answer choices

a) According to a fixed SOP (Standard Operating Procedure)
b) As instructed by the supervising physician
c) At the physician's own discretion

Q13. Which variables are included in your SOP for managing intraoperative hypotension? (Please select all applicable variables)

Answer choices

a) Cardiac index (l/min/m²)
b) Cardiac output (l/min)
c) Dynamic arterial elastance (Ea_{dyn})
d) Left ventricular contractility (dP_{max} or dP/dt)
e) Mean arterial pressure (MAP)
f) Passive leg raise test (PLR)
g) Pulse pressure variation (PPV)
h) Stroke volume (SV)
i) Stroke volume index (SVI)
j) Stroke volume variation (SVV)
k) Ultrasound of the vena cava inferior
l) Systolic pressure variation (SPV)
m) Systemic vascular resistance (SVR/SVRI)
n) Transoesophageal echocardiography (TOE)
o) Transthoracic echocardiography (TTE)
p) Central venous pressure (CVP)

Q14. Which variables would you prefer to use for therapy management? (Please select all applicable variables)
Answer choices

a) Cardiac index (l/min/m²)
b) Cardiac output (l/min)
c) Dynamic arterial elastance (Ea_{dyn})
d) Left ventricular contractility (dP_{max} or dP/dt)
e) Mean arterial pressure (MAP)
f) Passive leg raise test (PLR)
g) Pulse pressure variation (PPV)
h) Stroke volume (SV)
i) Stroke volume index (SVI)
j) Stroke volume variation (SVV)
k) Ultrasound of the vena cava inferior
l) Systolic pressure variation (SPV)
m) Systemic vascular resistance (SVR/SVRI)
n) Transoesophageal echocardiography (TOE)
o) Transthoracic echocardiography (TTE)
p) Central venous pressure (CVP)

Q15. Which variables do you prefer to use for therapy management? (Please select all applicable variables)
Answer choices

a) Cardiac index (l/min/m²)
b) Cardiac output (l/min)
c) Dynamic arterial elastance (Ea_{dyn})
d) Left ventricular contractility (dP_{max} or dP/dt)
e) Mean arterial pressure (MAP)
f) Passive leg raise test (PLR)
g) Pulse pressure variation (PPV)
h) Stroke volume (SV)
i) Stroke volume index (SVI)
j) Stroke volume variation (SVV)
k) Ultrasound of the vena cava inferior
l) Systolic pressure variation (SPV)
m) Systemic vascular resistance (SVR/SVRI)
n) Transoesophageal echocardiography (TOE)
o) Transthoracic echocardiography (TTE)
p) Central venous pressure (CVP)

Q16. Which criteria influence your decision to establish advanced haemodynamic monitoring?

	Always/ Frequently	Occasionally	Rarely/ Never
Answer choices			
a) ASA status			
b) Expected blood loss			
c) Comorbidities			
d) Duration of surgery			
e) Surgical risk			
f) Patient positioning (e.g., sitting position, prone position, lateral position, etc.)			

Q17. Which medications are generally available to you in the operating theatre for the treatment of intraoperative hypotension? (Multiple selections possible)

Answer choices

a) Adrenaline/Epinephrine
b) Cafedrine/Theodrenaline (Akrinor®)
c) Dobutamine
d) Ephedrine
e) Noradrenaline/Norepinephrine
f) Phenylephrine
g) Vasopressin
h) Other (please specify)

Q18. Which cardiovascular drug (administered via a peripheral venous cannula) do you most frequently use during anaesthesia induction to treat anaesthesia-associated hypotension in non-cardiac surgical patients to achieve the target blood pressure?

Answer choices

a) Adrenaline/Epinephrine
b) Cafedrine/Theodrenaline (Akrinor®) – Bolus doses
c) Cafedrine/Theodrenaline (Akrinor®) – Infusion pump
d) Dobutamine – Bolus doses
e) Dobutamine – Infusion pump
f) Ephedrine
g) Norepinephrine/Noradrenaline – Bolus doses
h) Norepinephrine/Noradrenaline – Infusion pump
i) Phenylephrine
j) Other (please specify)

Q19. Which blood pressure value do you use to guide your perioperative treatment of hypotension?

Answer choices

a) Systolic blood pressure
b) Mean arterial pressure
c) Diastolic blood pressure

Q20. Which systolic blood pressure value do you consider “critically low” in non-cardiac surgical patients, or at which systolic blood pressure value do you begin treatment?
Answer choices

a) 100 mmHg
b) 90 mmHg
c) 80 mmHg
d) Target is the systolic blood pressure measured immediately before induction of anaesthesia
e) Values that are ≥ 20% below the systolic blood pressure measured immediately before induction of anaesthesia
f) Target is the systolic blood pressure measured preoperatively (pre-assessment clinic, patient chart, outpatient measurement)
g) Values that are ≥ 20% below the preoperatively measured systolic blood pressure (pre-assessment clinic, patient chart, outpatient measurement)
h) Other (please specify systolic blood pressure value in mmHg)

Q21. Which mean arterial pressure (MAP) do you consider “critically low” in non-cardiac surgical patients, or at which MAP do you begin treatment?
Answer choices

i) 70 mmHg
j) 65 mmHg
k) 60 mmHg
l) 55 mmHg
m) 50 mmHg
n) Other (please specify MAP in mmHg)

Q22. Which diastolic blood pressure value do you consider “critically low” in non-cardiac surgical patients, or at which diastolic blood pressure value do you begin treatment?
Answer choices

a) 60 mmHg
b) 50 mmHg
c) 40 mmHg
d) 30 mmHg
e) Other (please specify diastolic blood pressure value in mmHg)

Q23. Which variable or measurement method do you prefer to use for evaluating volume responsiveness?
Answer choices

a) Pulse pressure variation (PPV)
b) Stroke volume (SV)
c) Passive leg raise test (PLR)
d) Stroke volume variation (SVV)
e) Ultrasound of the vena cava inferior (IVC)
f) Systolic pressure variation (SPV)
g) Central venous pressure (CVP)
h) Mean arterial pressure (MAP)

Q24. How do you primarily assess intraoperative volume responsiveness (increase in preload leads to an increase in stroke volume) in an invasively monitored and mechanically ventilated patient (with sinus rhythm)? (Please select up to 3 answers)

Answer choices

a) Assessment of capillary refill time
b) Pulse pressure variation (PPV)
c) Stroke volume variation (SVV)
d) Skin condition (oedema vs. skin folds)
e) Heart rate
f) Clinical/physician experience
g) Lactate
h) Plethysmographic amplitude variation
i) $ScvO_2$ or SvO_2
j) Trendelenburg position
k) Urine output per hour
l) IVC diameter and its respiratory modulation
m) Visual assessment of the arterial blood pressure waveform (“swing”)
n) Volume bolus (250 - 500 ml)
o) Volume bolus (100 ml)
p) CVP

Q25. To assess volume responsiveness, a defined fluid bolus (“fluid challenge”) can be administered over a short period, and a potential change in stroke volume is measured. Which fluid do you prefer to use?

Answer choices

a) NaCl 0.9%
b) Balanced full-electrolyte solution
c) Albumin
d) Hydroxyethyl starch (HES)
e) Gelatine
f) Dextran
g) Other (please specify)

Q26. What volume do you use as a fluid bolus for the “fluid challenge”?

Answer choices

a) 100 ml
b) 200 ml
c) 250 ml
d) 500 ml
e) Weight-adjusted (ideal body weight)
f) Other (please specify)

Q27. Which variable do you prefer to use to evaluate contractility?
Answer choices

a) Cardiac index (l/min/m²)
b) Left ventricular contractility (dPmax or dP/dt)
c) Stroke volume (SV)
d) Other (please specify)

Q28. Which variable do you prefer to use to evaluate afterload?
Answer choices

a) Dynamic arterial elastance (Eadyn)
b) Mean arterial pressure (MAP)
c) Systemic vascular resistance (SVR)
d) Other (please specify)

Q29. An acute blood loss with relevant hypotension occurs intraoperatively. You administer 750 ml of an isotonic electrolyte solution over 5 minutes. Which fluid do you prefer to administer afterward until blood products (RBC, FFP, PLT) become available?
Answer choices

a) Albumin
b) Balanced full-electrolyte solution
c) Dextran
d) Gelatine
e) Hydroxyethyl starch (HES)
f) NaCl 0.9%
g) Other (please specify)

Q30. In many hospitals, advanced haemodynamic monitoring (AHM) is not used in high-risk non-cardiac surgical patients. In your opinion, what are the main reasons for this? (You may select up to 3 answers.)
Answer choices

a) AHM is not sufficiently reliable
b) AHM takes up too much space in the OR
c) Establishing AHM is too time-consuming
d) Lack of experience in interpreting values
e) Too few monitors for AHM are available in the hospital
f) AHM is not available in the hospital
g) The benefit of AHM is considered too low
h) Monitors/consumables are too expensive
i) Other (please specify)

Q31. Do you believe that using AHM can improve patient care in the operating theatre?
Answer choices

a) (Almost) always
b) Sometimes
c) (Almost) never
d) Uncertain / I don’t know
